# Kinship and Social Behavior of Lowland Tapirs (*Tapirus terrestris*) in a Central Amazon Landscape

**DOI:** 10.1371/journal.pone.0092507

**Published:** 2014-03-26

**Authors:** Gabriela M. Pinho, Anders Gonçalves da Silva, Tomas Hrbek, Eduardo M. Venticinque, Izeni P. Farias

**Affiliations:** 1 Programa de Pós-Graduação em Ecologia, Instituto Nacional de Pesquisas da Amazônia (INPA), Manaus, Amazonas, Brasil; 2 Laboratório de Evolução e Genética Animal, Departamento de Biologia, Universidade Federal do Amazonas (UFAM), Manaus, Amazonas, Brasil; 3 Division of Marine and Atmospheric Research, Commonwealth Scientific and Industrial Research Organisation (CSIRO), Hobart, Tasmania, Australia; 4 School of Biological Sciences, Monash University, Clayton, Victoria, Australia; 5 Laboratório de Ecologia e Conservação da Biodiversidade, Centro de Biociências, Universidade Federal do Rio Grande do Norte (UFRN), Natal, Rio Grande do Norte, Brasil; North Carolina State University, United States of America

## Abstract

We tested the hypothesis that tapirs tolerate individuals from adjacent and overlapping home ranges if they are related. We obtained genetic data from fecal samples collected in the Balbina reservoir landscape, central Amazon. Samples were genotyped at 14 microsatellite loci, of which five produced high quality informative genotypes. Based on an analysis of 32 individuals, we inferred a single panmictic population with high levels of heterozygosity. Kinship analysis identified 10 pairs of full siblings or parent-offspring, 10 pairs of half siblings and 25 unrelated pairs. In 10 cases, the related individuals were situated on opposite margins of the reservoir, suggesting that tapirs are capable of crossing the main river, even after damming. The polygamous model was the most likely mating system for *Tapirus terrestris*. Moran's *I* index of allele sharing between pairs of individuals geographically close (<3 km) was similar to that observed between individual pairs at larger distances (>3 km). Confirming this result, the related individuals were not geographically closer than unrelated ones (W = 188.5; *p* = 0.339). Thus, we found no evidence of a preference for being close to relatives and observed a tendency for dispersal. The small importance of relatedness in determining spatial distribution of individuals is unusual in mammals, but not unheard of. Finally, non-invasive sampling allowed efficient access to the genetic data, despite the warm and humid climate of the Amazon, which accelerates DNA degradation.

## Introduction

The ability of individuals to change their behavior based on the recognition of kin is an important characteristic in the evolution mammalian social systems [1], [2]. For instance, individuals can form philopatric social groups based on kinship or disperse from the natal home range. The way individuals behave with respect to related individuals will affect how genetic diversity is distributed in space [3]. Building an understanding of how different species behave towards kin is crucial to our investigation of the evolutionary causes of mammalian social behavior [4].

A common social outcome mediated by kin recognition in mammals is the formation of philopatric social groups, in which a number of closely related individuals remain together at or near their natal site and display cooperative behavior [5]. Surprisingly, and so far little explored, philopatric social groups are found not only in gregarious species but also in solitary ones [6]–[8]. In solitary species, while there is a significant overlap between home ranges, individuals perform their daily activities alone [6]. This helps explain why such behavior has attracted little attention, as direct interactions occur infrequently, thus making it difficult to carry out observational studies. Observational studies are often further complicated by the structure of the environment and the species' activity pattern, which can further decrease the probability of observing interactions *in situ*.

To overcome the issues with direct observation, indirect approaches based on home range overlap have been used as a measure of sociality in solitary species [9]. Nevertheless, this still requires capturing, radio-collaring, and monitoring several individuals over a suitable time scale. However, large mammals are not easy to capture. An alternative approach is provided by analysis of genetic data obtained from non-invasively collected samples. Such samples can be used to identify individuals and infer the degree of relatedness among them [10]. By examining the spatial distribution of relatedness we can obtain valuable insights into animal behavior, which do not require capture and manipulation of animals [11]–[13].

Here, we present genetic data on the lowland tapir (*Tapirus terrestris*), a large solitary mammal, in order to test the hypothesis that lowland tapirs exhibit social behavior based on relatedness. The little we know about social behavior in this species comes from observation of overlap in home ranges, radio-collar data, and anecdotal accounts. Substantial home range overlap between individual tapirs has been found in a number of studies encompassing a range of different biomes [14]–[16]. Medici [16] did not find significant differences in percentage home range overlap across the three possible gender pairs: 43.2% for male-male, 33.4% for female-female and 34.9% for male-female range overlap. The extent of home range overlap suggests that *T. terrestris* may display some sort of social behavior (as defined in Waser & Jones [6]).

In addition to home range overlap, indications that tapirs display territorial behavior are provided by movement data and exclusion behavior. Tobler [15] found that individuals regularly walked along the borders of their ranges, possibly monitoring a territory. With respect to exclusion behavior, resident *T. bardii* individuals were observed attacking newly translocated individuals, suggesting territorial defense [17]. Tapirs may also use latrines as a way of marking territory boundaries, a common behavior among mammals [18], [19] (but see Ralls [20] and Rostain *et al*. [21] for alternative explanations for latrine use). The evidence of territorial behavior and home range overlap suggests that tapirs can recognize different individuals, reinforcing the possibility that this species exhibits social behavior.

Mating systems are also often intimately associated with social behavior and may influence the degree of territoriality in a species [6]. Currently, we lack data on the tapir's mating system. Observations reported by C. R. Foerster in studies of *T. bairdii* indicate that tapirs are likely facultative polygynous [16]. Overlap in territory among related females is expected under polygyny [6]. Hence, polygyny can lead to increased spatial autocorrelation in genes at small spatial scales relative to broader spatial scales [22].

The apparent capacity to change behavior based on individual recognition lead us to suspect that kin recognition may influence patterns of interactions in *T. terrestris*. We thus hypothesized that tapirs tolerate individuals from adjacent and overlapping home ranges if they are related. Based on this hypothesis we expect to find pairs of related individuals geographically closer than pairs of unrelated individuals. This hypothesis was proposed by Medici [16], based on both personal communications with C. R. Foerster, and the study of Tobler [15], in which a male and female, likely sibs, were observed sharing their parent's home range. According to C. R. Foerster [16], tapirs form family units, in which there are extensive home range overlaps between related individuals and non-related individuals are excluded. Our study is the first, to our knowledge, to test this hypothesis using genetic information.

Thus, the objectives of the present study were: (1) to analyze the spatial distribution of related individuals of *T. terrestris*; and (2) determine the species mating system.

## Materials and Methods

### Study area

The Balbina hydroelectric dam was flooded in 1987 and is located approximately 150 km north of the city of Manaus (Amazonas state, Brazil). Due to the flat topology, the reservoir has extended over 2360 km^2^ creating over 3500 islands [23]. To offset the environmental impact caused by the dam the Brazilian government created the Uatumã Biological Reserve in 1990 (0°50' to 1°55' S, 58°50' to 60°10' W). The Uatumã reserve is predominantly composed of continuous forest and its buffer zone includes the lake and island formations. The area is dominated by dense tropical rainforest with an average tree height of 30 meters [23].

Preliminary surveys conducted in the islands of the Uatumã Biological Reserve suggested a high density of tapirs (M. Benchimol personal communication). This was in stark contrast to our previous experience in continuous forest in the Jaú National Park (AGS), where tapir densities were low and dung samples were rare. The observation of greater densities of tapirs in seemingly disturbed habitat is not uncommon [24]. Also, the distribution across islands facilitates fieldwork, as a larger area can be covered by boat than what would be feasible on foot in the jungle. Furthermore, the footprints in the margin of the islands were important indicators of the presence of tapirs and, therefore, provided ample opportunities to collect dung.

**Figure 1:**
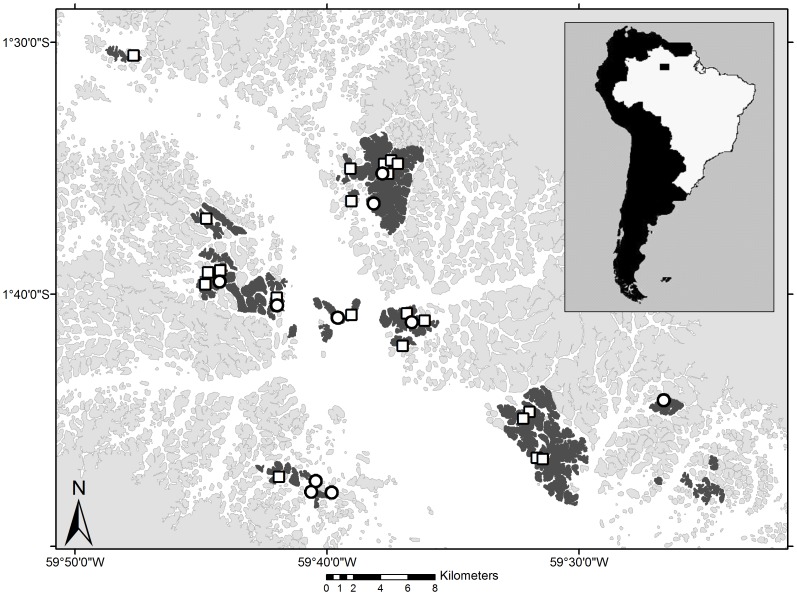
The Balbina hydroelectric dam reservoir, central Amazon, Brazil. Active search for feces was carried out on islands highlighted in dark gray. Open circles and squares represent sites at which the samples used for population analyses were found (N = 32). Only samples represented by open squares were used in kinship inference (N = 22).

The ability of tapirs to swim, the relatively small distance between adjacent islands (see Figure 1), the high tapir density and the logistical efficiency suggested that it would be more feasible to study tapir behavior on the islands than in areas of contiguous forest. Therefore we selected groups of islands (islands in close proximity to one another) to be sampled intensely in order to capture all tapirs, and ascertain that we have described all possible local relatedness connections. To cover a greater portion of the reservoir and have a representative sampling, we surveyed several groups of adjacent islands in different areas of the reservoir (Figure 1). We used Landsat TM5 satellite images from 2008 analyzed with ArcGIS 9.3 [25] to assist in the selection of target islands. A total of 48 islands were visited over a period of 55 days predominantly in the dry seasons of 2009 and 2010. Sampling was carried out under permit no. 21320-1 issued by the Instituto Chico Mendes de Conservação da Biodiversidade (ICMBio/MMA).

### Sample collection

A small aluminum boat with an outboard engine was used to circle the islands in search for recent signs of tapir activity and feces in the water. The number and age of footprints on the margin were determinants in the choice of landing sites for each island. We found a positive relationship between footprint density and the possibility of encountering latrines. Each landing lasted one hour, with three researchers actively looking for latrines or feces.

We only collected feces with a short exposure time (1 to 5 days). Disposable scalpels were used to scrape off approximately 1 ml of the fecal pellet surface. The sample was then placed in a Falcon tube (15 ml) containing 5 ml of Longmire buffer [26] or 10 ml of absolute ethanol. Sample and preservation buffer were thoroughly mixed by inversion to ensure a uniform mixture. The samples were maintained at ambient temperature throughout the fieldwork (maximum of 15 days) and preserved at −80°C in the laboratory.

DNA was isolated using the QIAmp DNA Stool Mini Kit (QIAGEN), following the manufacturer's protocol with the following modifications: (1) the initial amount of the sample was increased to 500 μl and (2) the final elution volume was decreased to 40 μl, as recommended by other authors [27],[28].

We used microsatellite markers previously developed for *T. terrestris*. Fourteen microsatellite loci were tested on blood samples from two captive tapirs held at the Mantenedor da Fauna Cariuá facility (Cadastro Técnico Federal– National Registry Number: 671958). Blood was drawn during scheduled veterinary care procedures by a registered vet (Laerzio Chiesorin Neto, CRMV 0284/AM) following standard procedures approved by the Brazilian Regional Veterinary Council, and the IUCN/SSC Tapir Specialist Group's Veterinary Committee (http://www.tapirs.org). The Animal Ethics Committee at INPA does not require prior approval to conduct sampling if it is deemed ‘prophylactic or for other veterinary care'. All care was taken to ensure that no animals suffered during the development of this study.

The tested primers were Tte05, Tte12, Tba20, TtGT070, TtGT137, Tte01, Tter4, Tter5, Tter7, Tter9, Tter13, Tter14, Tter17 and Tter18 [29]–[31]. The primers were labeled with a fluorescent dye using the method described by Schuelke [32]. The polymerase chain reaction (PCR) conditions were: 94°C for 1 min; 30 cycles of 94°C for 30 s, 52 or 56°C (depending on primer used) for 30 s and 68°C for 40 s; 30 cycles of 94°C for 20 s, 53°C for 30 s and 72°C for 1 min; followed by 72°C for 30 min and a final hold at 4°C. Genotyping was performed on an ABI 3130xl sequencer (Applied Biosystems), using a mix of 1 μl of the PCR product, 8 μl of formamide and 1 μl of the ROX size standard [33]. Allele size was inferred in the program GeneMapper v4.0 (Applied Biosystems).

Non-invasive samples are generally characterized by high rates of genotyping and amplification errors [34]–[36]. To ensure we only used high quality genotypes in analyses we used the multiple-tube approach [37]. In the multiple-tube approach, the genotype at a locus is determined by consistent observation of alleles across multiple PCRs. The exact number of PCRs largely depends on the amount and quality of DNA that can be obtained from the fecal samples, and the resources available to repeat PCR reactions. Taberlet *et al*. [37] suggest that an initial three positive PCRs be performed, and if ambiguity persists, another four PCRs should be carried out.

We modified the Taberlet *et al*. [37] approach in order to fit with our budget, sample, and laboratory constraints. We included in the final dataset only genotypes that were observed at least three times in a maximum of seven PCRs per marker per sample. Three positive PCRs with consistent genotypes was the minimum required by Taberlet *et al*. [37] to achieve 99% confidence in the observed genotype when the genotype is heterozygous. We also propose that the mislabeling of heterozygous individuals as homozygous was not a significant error in our dataset because we: (1) did not detect departures from Hardy-Weinberg proportions and null alleles; (2) re-captured genotypes in close geographic proximity; and (3) observed and expected heterozygosities did not differ significantly from other studies (see Results section).

We found that DNA degraded rapidly after isolation, thus, to obtain three consistent amplifications, an average of three extractions were needed per sample in order to obtain sufficient DNA for all the PCRs. Extractions were not pooled, but instead were performed as needed following PCR amplification failures. Genotypes from samples that did not amplify in five consecutive PCRs *per* primer were discarded.

The DNA extraction, PCR preparation and PCR handling prior to genotyping were all performed in different laboratories in order to avoid contamination. Sample preparation for genotyping was the only step carried out in a laboratory in which samples from other animals were present. PCR mixtures were prepared in a PCR hood sterilized with ultraviolet light. Positive and negative controls were included as part of each PCR batch and negative controls were included in the DNA extraction step. If the negative control was positive in the PCR, the control was genotyped and, if successful, the samples with similar alleles to the negative control were discarded.

### Data analysis

The genotypes were analyzed for the presence of null alleles, allelic dropout and stuttering using the program Micro-Checker v2.2 [38]. To measure the statistical power of the primer set for individual identification we calculated the probability of identity (*P*_(ID)_), which is the probability that two non-related individuals have the same genotype in a population [39]. The *P*_(ID)unbiased_ and the *P*_(ID)sib_ were calculated using Gimlet v1.3.3 [40], correcting for the number of sampled individuals and the possibility of sampling related individuals [41]. Arlequin v3.5 [42] was used to estimate heterozygosity and test for deviations from Hardy-Weinberg proportions and linkage equilibrium. Bonferroni correction [43] was applied to adjust statistical significance across multiple tests.

To test our hypothesis it was necessary to first establish the number of distinct genetic units sampled in the Balbina reservoir. The number of units was estimated using Structure v. 2.2 [44]. We allowed for the possibility of admixture and considered the allelic frequencies correlated between genetic units. One to four populations (*K*) were tested and *a priori* we considered all *K* to be equally likely. We did not use location information to inform the prior (i.e., uninformative prior). For each *K*, we ran 10 chains each with 10^6^ iterations, with the first 10^5^ iterations discarded as burn-in. The most likely *K* was inferred by maximizing the log-likelihood of the data given *K*. Convergence of the MCMC was assessed by visual inspection of chains within Structure, and by comparing results across multiple runs of Structure. We were satisfied that convergence was achieved when we did not observe any trends in the chains, and that results across chains were largely comparable. It is hard to assess with complete confidence that convergence has been attained, but it is usually easy to determine that convergence has not been reached [45]. In our approach, we reduce the uncertainty about the effect of the starting values on the final outcome, and we minimize the risk that inferences are being drawn on MCMC that have not yet reached stationarity [46].

We used ARLEQUIN to estimate gene diversity, and estimate *F_ST_* and *F_IS_* values [47] based on the analysis of molecular variance (AMOVA) between individuals on the eastern (*n* = 18) and western (*n* = 12) banks of the reservoir. We chose this grouping because we believe that the lake is the greatest potential barrier to dispersal in the reservoir landscape. Individuals that did not clearly belong to either margin were excluded from this analysis (*n* = 2).

We also estimated the effective population size (*N_e_*) using the program MIGRATE-N v2.1 [48]. We used Bayesian inference and maximum likelihood to estimate the parameter *θ*, which was subsequently converted into coalescent effective population size using the formula *θ* = 4*N_e_μ*. Since there is no estimate of microsatellite mutation rate (*μ*) for any of the species of tapirs, we considered a range of mutations rates from 1×10^−4^ to 5×10^−4^. These mutations rates encompass estimates used in mammalian studies [49]–[51].

To infer *θ* using maximum likelihood we ran 10 short chains, sampled each chain 5×10^4^ times and recorded 500 genealogies. We then ran three long chains, sampled each chain 1×10^6^ times, and discarded the first 1×10^5^ samples as burn-in. In the Bayesian inference analysis, we ran one long chain, which was sampled 5×10^6^ and recorded every 100^th^ genealogy. Searches were replicated 10 times. Search of genealogy space was improved via adaptive swapping among chains.

Estimates of relatedness between pairs of individuals can be highly variable, and different relatedness estimators will have distinct behaviors for any given dataset and particular relatedness category [52], [53]. In order to investigate the properties of different estimators given the observed allele frequencies in our dataset, we used Coancestry v.1.0 [54] to simulate 100 pairs of individuals in each of the four major relatedness categories (PO: parent-offspring; FS: full-sibs; HS: half-sibs; and UN: unrelated). Because of the difficulty in separating PO and FS pairs, we grouped this category into a single first-order (FO) relationship category. We then used Colony v2.0 [55], Kingroup v2.08 [56] and Identix v1.1 [57] to classify the simulated pairs into relatedness categories. We used the results from our simulated pairs as a training set in order to set expectations about classifying pairs of samples in our dataset.

In Colony, due to the lack of data on sex and age of the individuals, the same individuals were set as possible candidates for siblings, bulls and cows. We used a prior probability of 0.5 that at least one true cow or bull was present in the dataset and accepted only relationships with a greater than 50% probability of belonging to a relatedness class. Colony uses the information about the mating systems to perform the classification of pairs into relatedness classes. We estimated the likelihood of the data given three different breeding systems: (1) monogamy; (2) polygyny or polyandry; and (3) polygamy. We used Bayes factors [58] to identify which mating model had highest posterior support given the available data. Bayes factor values less than −2.0 (Log_10_ scale) were considered an indication of a significantly better fit of the more complex model to the observed data [58].

The relatedness index (*r*) of Lynch and Ritland (*r*_LR99_ [59]) and Queller and Goodnight (*r*_QG89_ [60]) were estimated with Kingroup and Identix. We used Identix to estimate the 95% confidence interval for each pairwise *r* by bootstrapping. We used Kingroup to test relationship hypotheses against more than one null hypothesis using likelihood ratio tests. In other words, we asked what is the likelihood odds ratio of a pair being PO given that FS, HS and UN are null hypotheses.

The results of the analyses for the simulated pairs were checked in R [61]. We calculated the proportion of unrelated pairs being classified as related (type I error), the number of related pairs being classified as unrelated (type II error), the proportion of first order relationships pairs (PO and FS) being misclassified as something else (misFO) and the proportion of UN and HS pairs being misclassified as first order relatives (misHS/UN). To assist the classification of some relationships in the tapir dataset, for each relatedness category we calculated the mean number of loci for which at least one allele was shared between a pair, and the mean number of alleles shared between individuals in a pair (see Table S1 for more details).

The classification of the simulated pairs suggested that Colony performed poorly with our dataset (see Results section). Thus, we combined the results of Kingroup, Identix, and allele sharing patterns in order to produce a final classification for each pair in our dataset. Based on the simulation results, we took the following conservative approach to classify individual pairs into relatedness categories:

(1) Based on the observed confidence interval surrounding an individual pair's *r_LR99_* and *r_QG89_*, we classified pairs as:

- *First-order relatives* (FO): if the lower bound of the confidence interval was ≥0.5;
- *Half-sibs/Firs-order relatives group 1* (HS/FO1): if the lower bound of the confidence interval was ≥0.25 and <0.5;
- *Half-sibs/Firs-order relatives group 2* (HS/FO2): if the lower bound of the confidence intervals was ≥0.125 and <0.25;
- *Unrelated* (UN): if the upper bound of the confidence interval was ≤0;
- *Inconclusive* (IN): the pair was classified as ‘inconclusive' if the confidence intervals was >0 and <0.125 or the confidence interval included zero and spanned over related classes.

(2) We accepted the likelihood ratio test with the lowest *p-value* among the following hypotheses comparisons:

- Parent-offspring vs full-sibs and unrelated;
- Full-sibs vs half-sibs and unrelated;
- Half-sibs vs cousins and unrelated;
- Cousins vs unrelated, and;
- Unrelated vs parent-offspring, full sibs, half sibs and cousins.

(3) We classified pairs as FO if they shared ≥7 alleles at ≥0.8 of the loci.

The results from our simulations found a large overlap among confidence intervals between *half-sib* (HS) pairs and *first-order relatives* (FO; parent-offspring or full-sibs). Thus, we created a HSFO category, which groups individuals that are likely related but we are unsure to what degree. As there were more FO relatives in the interval from 0.25≤*r*<0.5 than in the interval 0.125≤*r*<0.25, we decided to created to subgroups of HSFO. This allowed us to create a finer gradient for classification of relatedness than a single HSFO category ranging from 0.125≤*r*<0.5 would allow.

The relationships in the tapir dataset that could be assigned to a class were used to test for associations with geographic distances. Pairwise geographic distances were estimated as the Euclidian distance between individuals, and were based on geographic coordinates recorded for each sample (collected with a Garmin GPSMAP 60CSx). We tested if the geographic distances of related individuals (classified as first-order or half sibs) were smaller than the distance between unrelated ones using a Mann Whitney U test. We also performed a Mantel test to test for an association between observed *r* values and geographic distances. Both tests were carried out in R [61]. For the Mantel test, we used the *ncf* package [62].

Finally, Moran's *I*, an index of spatial autocorrelation [63], was estimated based on allele sharing at two scales: individual and landscape. Based on the mean home range described for *T. terrestris* [14]–[16], we assumed that samples distanced less than three km were deposited by individuals that likely have overlapping home ranges; we called this the individual scale. The landscape scale consisted of pair comparisons between samples separated by more than three km. We estimated 95% confidence intervals around estimates of Moran's *I* by bootstrapping individuals across both scales; this analysis was performed in SPAGeDi v1.3 [64].

## Results

Eleven of the 14 microsatellite markers amplified in the blood samples. One of these markers was monomorphic (Tter18) and five were sensitive to the low quality of DNA from fecal samples (TtGT070, Tte01, Tter13, Tter14, Tter9), resulting in either non-amplification or difficult to interpret electropherograms. The remaining five loci were used in kinship and population analyses, and formed our genotyping panel. No null alleles, allelic dropout, genotyping errors, linkage disequilibrium or deviations from Hardy-Weinberg proportions were detected in any of the five loci of the genotyping panel.

In spite of being able to genotype only five loci, these were sufficiently informative to discriminate individuals: we estimated a *P*_(ID)unbiased_ of 2.25×10^−6^ and a *P*_(ID)sib_ of 9.67×10^−3^, which are considered sufficiently stringent for conservation purposes (less than 0.01 [41]). Mean observed heterozygosity was 0.7721 and allelic diversity was 6.6 alleles/locus (Table 1).

**Table 1:** Microsatellites loci used for genotyping individuals of *T. terrestris*.

Locus	Motif	Size (pb)	*N*	*A*	*Ho*	*He*	*P*_(ID)unbiased_	*P*_(ID)sib_
Tte05	(TC)_10_(AC)_10_	143–159	31	7	0.77	0.78	0.077	0.390
Tte12	(AC)_19_	162–174	29	6	0.72	0.77	0.080	0.396
TtGT137	(GT)_17_	239–263	21	10	0.86	0.86	0.030	0.339
Tter4	(TG)_20_	237–245	25	5	0.88	0.81	0.063	0.374
Tter5	(GT)_10_	193–201	32	5	0.63	0.63	0.196	0.494
*Summary*		143–263	27.6 (±4.56)	6.6 (±2.07)	0.77 (±0.10)	0.77 (±0.08)	2.3e^−6^	9.6e^−3^

Note: motif type (Motif), allele size variation (Size), number of samples (*N*), allele richness (*A*), observed heterozygosity (*Ho*), expected heterozygosity (*He*), probability of identity with sample size correction (*P*_(ID)unbiased_) and probability of identity between sibs (*P*_(ID)sib_).

Approximately 1000 fecal samples were found, but only 63 were considered sufficiently fresh to sample. Among the samples collected for laboratory analysis, 10 amplified across three loci, two amplified across four loci and 24 amplified across all five loci. Only 20 genotypes were unique across the five loci, and four genotypes were repeated once across samples. This suggests that feces for each of four individuals were collected twice. The samples with replicate genotypes were collected within a two-day interval and were separated by 630, 505, 400 and 150 m.

Samples collected in water were useful for genetic analysis: 58% (14) of the samples that amplified at five loci were collected in water. Rapid degradation of the extracted DNA was observed for all samples even when kept at −80°C, with amplification failing approximately 15 days after extraction.

We used the 32 unique genotypes (with a minimum of three loci) for population analysis. In Structure, the maximum marginal log-likelihood of the data given *K* (logL(D|K)) was found for *K* = 1. This suggests the presence of a single genetic unit in the study area, which is corroborated by the AMOVA results between opposite margins of the reservoir. Most of the genetic variance was contained within each margin rather than between margins. This resulted in low *F_ST_* (0.008) and *F_IS_* (−0.011) values (both *p>*0.05). The gene diversity index for the population of the Balbina reservoir was 0.6634±0.4207.

Bayesian and maximum likelihood estimates of *θ* obtained with Migrate-N were 6.5 (95% HPD 3.67–9.26) and 7.1 (95% CI 6.1–8.10), respectively. These values correspond to coalescent effective population sizes ranging between 16250 and 3250, and 17750 and 3550 individuals, respectively, assuming a mutation rate of 1×10^−4^ and 5×10^−4^, respectively.

As shown in Table 2, errors surrounding estimates of *r*, the relatedness index, in the programs Kingroup and Identix were similar, and the type I errors were relatively high (0.32). The full-pedigree likelihood method implemented in Colony was extremely conservative, with a great number of first-order pairs being misclassified (misFO = 0.94). The small sample size, marker number and the lack of information about the individuals (sex and age) probably affected the classifications made by Colony. Also, Colony does not identify UN pairs, thus we considered all unclassified pairs as UN; this likely has inflated our Colony estimates of misFO and type II error.

**Table 2:** Errors associated with relatedness estimates based on the results for the simulated population.

Software	Test	Type I	Type II	misFO	misHS/UN
Kingroup	*r*_QG89_	0.32	0.06	0.10	0.37
	*r*_LR99_	0.32	0.07	0.10	0.28
	hypothesis test	0.04	0	0.38	0.04
Identix	*r*_QG89_	0.32	0.06	0.11	0.37
	*r*_LR99_	0.31	0.07	0.10	0.28
	CI_LR99_	0.03	0.01	0	0.07
Colony	full-pedigree method	0.03	0.74	0.94	0

Note: Queller and Goodnight's *r* (*r*_QG89_), Lynch and Ritland's *r* (*r*_LR99_), pedigree hypothesis test, Lynch and Ritland's *r* confidence interval (CI_LR99_), the proportion of first order relationships being misclassified (misFO) and the proportion of pairs being misclassified as first order (misHS/UN).

Kingroup's pedigree hypothesis test was also conservative, but it had a smaller misFO error (0.38) and smaller errors overall than those observed with Colony. Moreover, the confidence interval estimates, analyzed in Identix, was the most reliable method: all errors were below 0.1 (Table 2). The main reason for these smaller errors was the classification as “inconclusive” for confidence intervals that included values between 0 and 0.125. Furthermore, allele sharing patterns proved to be a good approach to classify FO pairs: 87% of simulated pairs that shared ≥7 alleles at ≥0.8 of the loci were FO pairs.

Due to potential problems associated to missing data in kinship analyses, we restricted our analysis to samples that amplified for four (*n* = 2) or five (*n* = 20) loci. The polygamy model had the most support from the data, with the other two models obtaining Bayes factor values less than −20.0 (Table 3). Based on the above-described criteria to classify relationships, we found 10 first order relationships (parent-offspring or full sibs pairs – FO), 10 half-sib relationships, 25 unrelated pairs, and 186 inconclusive pairs.

**Table 3:** Maximum log-likelihood values for mating system models suggested for *T. terrestris*, with associated log Bayes factor and posterior probabilities.

Mating system	Log likelihood	Log Bayes Factor	Posterior Probability
Polygamy*	−796.38	0.00	1.00
Polygyny or Polyandry	−809.17	−25.6	7.6×10^−12^
Monogamy	−815.89	−39.04	1.01×10^−17^

*Model with highest posterior support

In five first order relationships and five half-sib pairs, the individuals were located on opposite sides of the reservoir. The distance between FO individuals ranged from 0.1 to 29.9 km (median: 9.4; lower quartile: 4.5; upper quartile: 13.0), for half-sib pairs ranged from 1.1 to 15.0 km (median: 11.3; lower quartile: 9.5; upper quartile: 14.8) and between unrelated individuals ranged from 0.5 to 23.3 km (median: 11.5; lower quartile: 9.0; upper quartile: 17.2). We did not find a statistical difference between distances of related pairs and unrelated pair distances (W = 188.5; *p* = 0.339) or between FO pair distances and unrelated pair distances (W = 77.5; *p* = 0.225).

Geographic coordinates were available for 21 samples of the 22 used for kinship analysis, resulting in 210 pairs. The Mantel test showed a non-significant and slightly negative correlation between the *r* value and the geographic distances (Queller and Goodnight's *r*: −0.102, *p* = 0.079; Lynch and Ritland's *r*: −0.113, *p* = 0.054). Moran's *I* at the individual scale (−0.0064±0.0558, number of pairs = 30) also did not differ from that of the landscape scale (0.0003±0.0096, number of pairs = 180).

## Discussion

In this study, we present non-invasive genetic data on *T. terrestris* sampled from the islands formed by the Balbina hydroelectric reservoir in central Amazon. Our objective was to test the hypothesis: individuals that overlap in their home ranges are more likely to be related than individuals that do not overlap in their home ranges. Below we interpret our results in terms of what we were able to achieve logistically, and what our results mean for tapir biology and mammal social behavior.

Finding fecal samples suitable for genetic analysis in the Amazon rainforest is hindered by the dense forest and by the region's climate. The dense canopy results in relatively dark understory, while leaf litter act as camouflage, making it difficult to spot dung samples. Meanwhile, the warm, humid climate accelerates DNA degradation in feces [65], [66]. The local environmental conditions notwithstanding, once samples were found that were considered sufficiently fresh for analyses, storage time became an important factor influencing amplification success. Several samples were collected while optimization of laboratory protocols was still underway, which resulted in longer storage time and lower genotyping success rate. Thus, we recommend that protocols be established and optimized prior to initiating fieldwork [34].

Despite these operational difficulties, we were able to obtain a reasonable number of samples for a large Neotropical mammal. In 55 field days we obtained reliable information on at least 20 individuals, as identified by genotype profiles. In comparison, studies based on animal capture, such as those of Tobler [15] and Medici [16], caught seven individuals over a six month period in the Peruvian Amazon and 35 individuals over the span of approximately nine years in the Atlantic Forest, respectively. It is also apparent that non-invasive samples can be used for recapture studies in the Amazon biome; samples with identical genotypes were collected within a short span of time and at close distances, which increases our confidence in a true recapture. Thus, the use of non-invasive sampling allow relatively rapid access to important biological information about elusive species [67], and provide encouragement for future research on elusive tropical species.

A large proportion of the samples were collected in water rather than on land. Contrary to general expectation, our results demonstrate that dung samples found in water bodies in tropical terrestrial ecosystems can yield high-quality genetic data. The lack of strong water currents in the Balbina reservoir and sampling during the dry season allowed the feces to remain intact for a greater period of time. This opens up the possibility of sample collection in study areas that encompass rivers without a strong current or in lakes. It should be noted that a sample in water that was carried by the wind/current could be differentiated from defecation at the collection sites by the appearance, quantity and grouping pattern of the pellets.

Our results indicate a single genetic unit in the landscape of the Balbina reservoir. While this result is important in regards to validating the assumptions of the relatedness analyses, it is also an interesting result in terms of landscape genetics. It suggests that the Uatumã river does not act as a barrier to gene flow in *T. terrestris*. However, the question of whether the increased width of the Uatumã river will have an effect is not likely to be answered any time soon. The time elapsed since the damming of the river (24 years) has not been sufficient relative to the species generation length to generate large effects on the spatial distribution of genetic variation (e.g.[68]): the life expectancy of tapirs in captivity is 30 years [69] with a generation time of approximately 11 years [16]. Thus, the low *F_IS_* and *F_ST_* values more likely reflect values of gene flow and genetic diversity that existed prior to the flooding of the dam. Hence, our results may be used as a benchmark in future studies aimed at assessing potential disturbances caused by the building of the dam.

However, we argue that our data carries evidence that demonstrates that the width of the lake does not pose a complete barrier to tapir movement in this landscape. Assuming a typical life table for mammals [70], with high mortality rates among juveniles and adults at an advanced age, the proportion of individuals in the population as old or older than the dam is probably less than 5%. Moreover, the life expectancy of mammals in the wild is generally lower than that of those kept in captivity. In this context, we feel comfortable in concluding that some of the 10 related pairs of individuals located on opposite margins of the reservoir include individuals born after the flooding. This corroborates the idea that barriers to gene flow (natural or artificial) in lowland tapirs occur at larger spatial scales that straddle more salient barriers, such as the Amazon River [71]. It is not possible, however, to say whether tapirs are able to swim across the full extent of the reservoir, or if islands along the old riverbed (Figure 1) are used as stepping-stones.

Bayesian (6.5) and maximum likelihood (7.1) estimates of *θ* (mutation scaled effective population size) were similar, as is expected when using non-informative priors. Given mutation rate assumptions, the effective population sizes may vary from 3250 to 17750. Generally the ratio of effective population size to census population size is thought to be around 1∶10. Thus the number of individuals in the Balbina reservoir region is large, ranging between 177500 and 32500 depending on the assumed mutation rate. If one considers that the census population for tapirs in an Atlantic Forest fragment of 360 km^2^ is ∼300 individuals [16], we would need an area ∼3 times larger than the REBIO Uatumã to harbor 30 thousand tapirs. This is not unreasonable given the continuity of the habitat in the region. Therefore, the estimated values are plausible if we consider that the geographic area occupied by the Balbina population is likely to be much larger than the sampled area.

The geographic extent of the population that includes Balbina is likely to be very large, considering that De Thoisy *et al*. [71], [72] demonstrated minimal differentiation and concomitantly high gene flow for *T. terrestris* over an area at least 100 times larger than that sampled in this study. As a further comparison Drummond *et al*. [73] and Spong *et al*. [51] estimated even larger effective population sizes than the present study for the Beringian bison and the Tanzanian leopard, respectively. Moreover, the estimated mean observed heterozygosity and allelic diversity in the present study are among the highest reported for large mammals [31]. De Thoisy *et al*. [72] found similar values and Gonçalves da Silva *et al*. [74] found slightly lower values for tapirs in captivity in Argentina (Table 4). For *T. bairdii* [31], the reported observed heterozygosity and allelic diversity values were considerably lower (Table 4), as expected for endangered populations [75].

**Table 4:** Studies that measured tapir genetic variability from microsatellite markers.

Species	*Ā*	Mean *Ho*	*L*	*N*	Material Collected	Study area	Reference
*T. terrestris*	6.6	0.77	5	32	Feces	Central Amazon	present study
*T. terrestris*	5.0	0.67	10	41	Blood	Argentina	[74]
*T. terrestris*	8.0	0.76	5	37	Tissue	French Guiana	[72]
*T. bairdii*	3.8	0.39	6	33	Hair/Tissue/Blood	Central America	[31]

Note: allelic diversity (allele/locus, *Ā*), mean observed heterozygosity (mean *Ho*), number of loci (*L*), number of individuals (*N*), type of material collected, and study area. None of the studies reported significant differences between expected and observed heterozygosity.

The mating system analysis we carried in Colony suggests that lowland tapirs are polygamous (Table 3). However, C. R. Foerster, after 10 years of study, suggested polygyny for Baird's tapirs (*T. bairdii*) [16]. With either mating strategy there is generally a high degree of home range overlap among adults, as found for *T. terrestris* [14]–[16], but the observation of home range overlap between one female with two males and of one male with two females [15], [16]—plus observations made by camera trapping of females being accompanied for different males (E. P. Medici, personal communication)—suggests a polygamous system (i.e., both male and female are promiscuous [76]). Therefore, the evidence found in the present study, together with ecological observations, support the hypothesis of a polygamous mating system for *T. terrestris*.

In general, polygamous ungulates that display some kind of territoriality are largely folivores observed in open-habitat areas, such as grasslands [77]. While our result would appear to contradict this observation, we do not believe it is entirely inconsistent with it. Instead, we propose that, if lowland tapirs are indeed promiscuous, the observation of a behavior typical of grassland habitat is a case of Krumbiegel's rule, which states that behavioral patterns evolved in one type of habitat will persist long after that habitat changes [78]. We know that tapirs in Asia evolved largely in open grasslands, and are now one of the few remaining taxa from a large megafauna that has not gone extinct with the rise of tropical jungles [79]. It is possible that similar scenario occurred in South and Central America [80].

Regarding the relatedness analysis, we classified pairs into a specific relatedness category based on the estimated *r*-values, confidence intervals surrounding each *r* estimate, pedigree hypotheses tests, mean number of shared alleles, and mean number of loci that share at least one allele (Table S1). As can be seen from our simulated data, the combined results increased our confidence in our classification, while accounting for the uncertainty resulting from the number of successfully assayed markers. Although we are confident that our classification is reliable, it is important to note that our sample sizes for the purpose of statistical analyses were small, as is the case for many studies with large mammals. Nevertheless, we consider the results informative and valuable, being the first data obtained via non-invasive sampling to identify individuals of an elusive mammal in the Amazon.

In the case where at least one of the sexes is philopatric there is an expectation of increased Moran's *I* at the local/social scale relative to larger, landscape scales (e.g. [81]). Our data show no difference between Moran's *I* at the individual scale and at the landscape scale. Pairs of related individuals did not occur geographically closer than pairs of unrelated individuals. We thus have no evidence to support the hypothesis that recognition between related individuals leads to a greater tolerance among tapirs, that tapirs prefer to be close to relatives or have philopatric behavior. Therefore, our data do not corroborate the formation of family units in *T. terrestris*.

The fact that kinship does not seem to influence the spatial pattern of individuals is unusual in mammals [82]. We are aware of only one example of this in mammals, the racoon (*Procyon lotor*) [83]. Interestingly, racoons and tapirs seem to have a lot in common. Much like tapirs, racoons are described as largely solitary wide-spread species, that occupy various types of habitats with varying densities. Similarly to our study, Hirsh *et al*. [83] found no pattern in spatial proximity between related and non-related individuals. Instead, they found that other factors, such as availability of winter dens and concentration of food resources played a much more significant role in driving associations between individuals. Thus, as in racoons, recognition between individuals may occur independently of kinship and other factors may influence the formation of social groups, such as environmental factors. Barongi [69] and Foerster & Vaughan [84] attributed tapir home range overlap to the fruiting season, in which the greater availability of food resources promotes group formation. It is also possible that the formation of the Balbina reservoir has disrupted territories and family units, and due to the long-lived nature of the species these characteristics have not yet returned to equilibrium.

The presence of unrelated pairs at the individual scale, coupled with the absence of correlation between relatedness indices and geographic distances suggests a high variance in tapir movement, which may represent dispersal events. Note that dispersal distance is defined as the distance between natal and breeding sites [6]. We cannot, however, distinguish between natal dispersal and breeding dispersal as we have no data for the ages of the animals studied. Adult individuals have been seen leaving their habitual home ranges by as much as 10 km to visit mineral licks [15]. So it is possible that adults make similar excursions in the search of the opposite sex.

Nevertheless, it is generally observed in mammals that individuals disperse from their natal site at the onset of sexual maturity, or soon after, to establish their own home ranges [5]. Foerster & Vaughan [84] observed the birth of four tapirs that dispersed from their natal area after three to four years. During the period of residence of the juveniles, their parents maintained an exclusive area without other adults. In this case, the establishment of territories would be associated with a period of parental care—but it is not clear, however, whether or not tapirs display territorial behavior. Thus, the observed high variance in distances between related individuals could reflect different stages of dispersal (e.g., before and after natal dispersal), as well as breeding dispersal or movement behavior associated with the search for resources.

The present study offers novel information on the behavioral ecology of *T. terrestris* and the use of non-invasive sampling for individual discrimination in tropical forests. Based on the present findings, we suggest a polygamous mating system and dispersal from the natal home range for *T. terrestris*. Apparently, tolerance between individuals is not influenced by kinship, as the proportion of related pairs at the individual scale was not different from the proportion observed at the landscape scale. This is unusual in mammals, but has been described elsewhere. In respect to the methods, the non-invasive sampling allowed rapid access to genetic data from an elusive species, even in the Amazon biome with its warm and humid forests. Therefore, the methods applied here should work for other medium-sized and large mammals in similar environments. However, researchers working in this perspective should be rigorous not only in the laboratory procedures, but also in testing kinship category assignments and selecting the most appropriate analytical methods for their data.

## Supporting Information

Table S1 **Information used in the classification of relationships.** Note: individuals in the pair (Ind1, Ind2); mean number of loci for which at least one allele was shared between a pair (Mean share); mean number of alleles shared between individuals in a pair (Allele count); relatedness index (*r*) of Lynch and Ritland (*r*_LR99_) and Queller and Goodnight (*r*_QG89_); 95% confidence interval for each pairwise *r* of Lynch and Ritland (CI_LR99) and Queller and Goodnight (CI_QG89); pedigree hypotheses test with the primary hypotheses being: parent-offspring (PO), full sibs (FS), half sibs (HS), cousins (C) and unrelated (UN); final classification (Conclusion) of a pair as inconclusive (IN) or into a relationship class (FO–First Order Relatives, HS–Half Sibs or U–Unrelated); geographic distance between the individuals in meters (Distance); and additional information used to assist the classification (Additional information). In the pedigree hypotheses test the tests with “*” were significant at the 0.05 level, “**” at 0,01 level and “***” at the 0.001 level. The values of distance with “†” symbol means that the individuals were located in opposite margins of the reservoir. The probabilities mentioned in the additional information were based on the errors measured from the results of the simulation.
(XLS)
